# The Sterilisation of Water for Troops in the Field

**Published:** 1908-11-28

**Authors:** 


### The Royal Army Medical Corps Section.

### THE STERILISATION OF WATER FOR TROOPS IN THE FIELD.

There is no more difficult problem, as the South African and other wars have shown, than that of providing a regular and sufficient supply of drinking water for troops on active service, whether in camp or on the march. Whatever system be adopted, it is essential that it should allow of such water being easily obtained, as otherwise the thirsty soldier will have recourse to whatever water is nearest at hand, notwithstanding the dangers attendant on doing so. Although these have been pointed out to him, and he has been trained to boil in his mess-tin water from any doubtful source, he cannot refrain from quenching his thirst, for he knows that boiling takes time, and leaves the water when cooled very insipid. Further, on active service there are very often no facilities for boiling water.

Although a good deal of thought and investigation have been devoted to this question of the best means of providing sterilised water for troops on active service, there is as yet no general unanimity on the point. All the usual methods of water purification by heat, filtration, and chemical means have been tried, but the experience of other nations, and of campaigning under varying conditions, have shown that they all have their disadvantages and impossibilities under certain circumstances. After the South African war our military authorities took the matter up, and in connection with it two very interesting demonstrations were given at Millbank Barracks in the spring and autumn of 1905. These indicated that the mechanical exclusion of germs by filtration was in all probability the most satisfactory and convenient plan of purifying water for the use of troops in the field. As an outcome of this decision there has been devised a field-service filter cart, called "the Brownlow patent filter cart," because the active agent in the filtration of the water is two series of Brownlow's unglazed porcelain candles or filter-tubes. Several of these filter carts were issued last camping season to the Territorial Force, and they are likely to be so again in future; so that all regimental medical officers should have a thorough knowledge of their construction and working, especially as their management is one of the duties of the water *personnel* of each unit.

In its present shape the Brownlow filter-cart consists of an iron tank, of a capacity of 110 gallons, mounted on two wheels and furnished with shafts to allow of its being drawn by animal traction. Externally this main water tank is furnished with two pumps and two clarifying filters of compressed sponge placed horizontally one on each side, while internally, as being the safest position for them, are fitted eight Brownlow candles for sterilising the water. These are arranged in two sets of four, in separate chambers, and each candle is covered with a special filter cloth to diminish any tendency to clogging. Although the pumps are independent, each pump serving the clarifying and sterilising filters on its own side, they can also be worked simultaneously. Beyond a small seven-gallon tank at the back of the cart there are no special arrangements for storing sterilised water, as it has been found that the filter-cart, when in proper working order, can sterilise water as fast as it can be distributed. To allow of rapid distribution the tank is fitted with twelve taps, four belonging to the seven-gallon tank of sterilised water and four to each set of the Brownlow candles, each candle having thus its own delivery tube. At these twelve taps the men's water bottles can be quickly and easily filled. At the front of the cart is a locker for holding spare parts, and strapped on to it is a kettle for sterilising the candles or filter-tubes. Coiled round hooks on the top of the main tank are two lengths of hose, which have at one end screw wing nuts for attaching them to the tank and at the other end roses fitted with wire gauze mesh. The hoses are for filling the main tank, and the roses are placed in the source of the water supply, the wire gauze keeping out mud and weeds.

The working of the filter cart is not a difficult or laborious matter, but it needs attention to details, and all medical officers should make themselves acquainted with these, especially as there are different procedures that may be adopted, each of which calls for a special arrangement of the mechanism. Thus it may be decided to filter the water direct from its source through the filters, or, on the other hand, to fill the main tank first with water clarified by passing through the filters of compressed sponge, and then to pass this water through the sterilising filters. This latter plan is the more usual, for the use of unclarified muddy water is very apt to clog the filter tubes and interfere with the working of the cart. In fact, if circumstances allow, as in camps, a preliminary clarification by alum or any of the other methods of rough filtration should be carried out at the source of the water supply if it is very muddy, as it gives the clarifying filters of the cart less to do, and facilitates the sterilisation through the Brownlow candles or filter tubes. Under ordinary circumstances, with a suitable supply of water, the main tank should be filled in half an hour or less, and in the first hour the cart should supply 100 gallons of sterilised water, but this amount should subsequently be greatly exceeded. Indeed, when being specially tried it was found after nine days' continuous use that the yield of sterilised water was 210 gallons per hour, an output far in excess of what is possible by any other method. This fact stamps the cart as a most valuable addition to the sanitary equipment of an army, while it has the further recommendation that it is to replace the existing objectionable water-cart allotted to each unit.

Satisfactory, however, as the Brownlow filter-cart is, its successful working depends on its being in the hands of men who understand its construction, so that it is very properly placed in charge of the water squad of the unit. Its transport should also be on regimental establishment, for filter carts are often needed at short notice and uncertain hours, and the Army Service Corps cannot always supply the horses needed when called upon. Another point requiring attention is that the horse drawing the cart should have saddlery that would allow of its being utilised to give assistance to the cart should it be in difficulties.

Experience in the field with these filter carts has furnished some points of practical importance. When issued from store they are often dirty inside and the taps stiff. They should be examined on arrival as to both these points. The pumps, too, need care and attention. They should be worked quietly and steadily, for they are not strong, and they are very liable to get out of working order. In a manner difficult to explain they are subject to stoppages, so that no water can be drawn through the hose pipes and the carts will not fill. Both the sponge and candle filters work satisfactorily, but the latter need very careful handling. A good deal of water is often lost from the tank behind, owing to the cover not fitting properly, and from the taps owing to the washers rotting. It is advisable to carry in the front locker a sufficient extra supply of these small leather washers. It is very necessary, too, to have a periodical cleaning and sterilising of the cart and its filters. The large tank should be flushed out once a fortnight and the small one weekly. Every three days the filter candles, with their cloths, should be placed in cold water in the kettle and the water raised to the boiling point, while every sixth day the filter cloths should be removed, rinsed in warm water, and the candles scrubbed on their surface with the brush supplied. After being replaced in their cloths the candles are boiled, and are once more fit for use. In the case of the clarifying filters their compressed sponges are rinsed weekly in boiling water and boiled once a fortnight. By observing these details the efficiency of the filter cart is ensured for any length of time, and there is no doubt that by its aid, coupled with the sterilisation from time to time of the water bottles of officers and men, which are readily contaminated under ordinary conditions of field service, the purity of the water supply of all the units should be assured, and protection afforded against water-borne diseases.

